# Complementary and integrative medicine use in mental disorders: socioeconomic patterns and healthcare utilization in Germany

**DOI:** 10.3389/fmed.2026.1904690

**Published:** 2026-08-21

**Authors:** Julia Siewert, Luca Thiel, Hannah Wackermann, Katja Icke, Michael Teut, Thomas Reinhold, Benno Brinkhaus, Nicolas Volz

**Affiliations:** 1 Institute of Social Medicine, Epidemiology and Health Economics, Charité—Universitätsmedizin Berlin, Corporate Member of Freie Universität Berlin and Humboldt- Universität zu Berlin, Berlin, Germany; 2 Institute of Social Medicine and Epidemiology, The Brandenburg Medical School Theodor Fontane, Neuruppin, Brandenburg, Germany

**Keywords:** complementary and integrative medicine, health economics, mental health, mental disorder, out-of-pocket expenditures

## Abstract

**Background:**

Complementary and integrative medicine (CIM) is widely used among individuals with mental disorders, but evidence on associated out-of-pocket expenditures and socioeconomic differences in spending remains limited.

**Methods:**

This cross-sectional study used data from the PSYKIM cross-sectional study of adults with self-reported psychiatric disorders in Germany. Associations of CIM-related out-of-pocket costs and expenditure categories with sociodemographic and clinical variables, physician awareness, subjective evaluation of CIM approaches, and health insurance status were examined descriptively and using multivariable binary and ordinal logistic regression models where appropriate. A relative financial burden measure, approximated as the ratio of CIM-related out-of-pocket expenses to available monthly income, was analyzed descriptively.

**Results:**

Among 657 participants with complete cost data, 72% reported out-of-pocket CIM-related costs. Higher disposable income was associated with higher odds of reporting out-of-pocket costs (aOR = 1.19; 95% CI: 1.04–1.37) and higher expenditure categories (aOR = 1.18; 95% CI: 1.06–1.31). Physician awareness of CIM use and more positive evaluations of CIM were also associated with higher expenditures. Men had lower adjusted odds of reporting out-of-pocket costs than women (aOR = 0.44; 95% CI: 0.26–0.76), while participants with university-level qualifications more frequently reported such costs in descriptive analyses. Disease duration, psychiatric diagnosis, and health insurance status were not clearly associated with CIM-related costs. CIM use was not associated with lower use of conventional medication.

**Conclusion:**

CIM use among individuals with mental disorders in Germany is frequently associated with private out-of-pocket expenditures and appears to be socially patterned. The findings further suggest that CIM is primarily used as an adjunct to, rather than a substitute for, conventional psychiatric treatment. Given the cross-sectional design, these observations should be confirmed in prospective observational studies.

## Background

Mental disorders represent a major global public health challenge and are associated with substantial individual, societal, and economic burdens. Based on estimates by Arias et al., building on data from the 2019 Global Burden of Disease study, approximately 418 million disability-adjusted life years (DALYs), corresponding to 16% of global DALYs, were attributable to mental disorders. The same study estimated the associated economic burden at approximately USD 5 trillion (4%−8% of regional gross domestic product [GDP], depending on the region) (1). A systematic review including 143 cost-of-illness studies from 48 countries further showed that mental disorders create a substantial economic burden across healthcare systems worldwide. The review found that indirect costs, particularly productivity losses, accounted for nearly half of the overall societal costs associated with mental disorders (2).

In Germany, mental disorders are both highly prevalent and economically significant. A nationally representative population-based survey reported a 12-month prevalence of 27.7%, indicating that more than one in four adults in Germany is affected by at least one mental disorder each year (3). Furthermore, a cost analysis based on the RECOVER trial in Hamburg found mean total 6-month excess costs of €19,075, with indirect excess costs (€13,331) substantially exceeding direct excess costs (€5,744) (4). These findings illustrate that mental disorders create not only a considerable clinical burden but also a substantial economic impact at both the individual and societal level.

Beyond the clinical burden of mental disorders, increasing attention has been directed toward the financial burden experienced by affected individuals. Out-of-pocket (OOP) payments constitute an important component of healthcare financing, even in countries with broad healthcare coverage. Such expenditures may arise through co-payments or cost-sharing (5). In Germany, a nationally representative survey found that 30% of adults with a need for medical care reported forgoing needed care in the previous year. Among these respondents, 11% identified financial costs as a reason for forgone care, with this pattern occurring more frequently among lower-income groups (6). The financial burden of OOP payments is especially relevant for people with chronic conditions. A European study across Germany, Belgium, and the Czech Republic found that several chronic diseases, including emotional disorders, were associated with higher OOP burden and that this burden was unevenly distributed among older adults with chronic diseases (5). These findings are particularly relevant for healthcare services that are not fully covered by statutory health insurance, such as complementary and integrative medicine (CIM), where patients may face out-of-pocket payments due to limited reimbursement.

From a health-economic perspective, CIM in Germany is often financed through out-of-pocket payments. Jeitler, Ortiz (7) note that many participants supported broader insurance coverage for CIM services, suggesting that reimbursement by statutory health insurers is currently perceived as insufficient. A multi-center survey at German university hospitals found high levels of CIM use among surgical patients, with 44% reporting current CIM use and many patients expressing strong interest in CIM counseling (8).

At the country level, higher health expenditure per capita is positively associated with greater CIM use, partly reflecting differences in reimbursement and integration of CIM into national healthcare systems (9). In Germany, 69% of adults stated that CIM costs should be covered by health insurance companies (7). Household income was also a significant predictor of CIM use. The highest utilization rate (38.2%) was observed among participants with a monthly household income of €4,000–5,000, compared to 19.6% among those with a household income of up to €500 (7). This points to a clear socioeconomic gradient in access to CIM healthcare. Against this background, self-financed CIM use may be particularly relevant for people with mental disorders.

Despite growing international evidence on CIM-related expenditures, little is known about the economic dimensions of CIM use among psychiatric patients in Germany. International studies show that people with mental disorders frequently use CIM alongside conventional treatment (10). In terms of self-financed expenditures, a US study found that adults with neuropsychiatric symptoms, including depression, anxiety, and insomnia, were significantly more likely to spend money on CIM than those without such symptoms, with total out-of-pocket expenditures in this group estimated at USD 14.8 billion (11). In Australia, annual out-of-pocket expenditures for CIM among adults with a self-reported mental health diagnosis amounted to approximately AUD 1.18 billion (12).

However, comparable German data on self-financed CIM expenditures among psychiatric patients remain scarce. To the best of current knowledge, no study has systematically examined the magnitude, distribution, or determinants of self-financed CIM expenditures among psychiatric patients in Germany. Against this background, the present study aimed to address this gap by examining CIM-related out-of-pocket expenditures among individuals with mental disorders in Germany and by exploring associations with socioeconomic factors, diagnostic groups, illness duration, perceived benefit, physician awareness, and conventional medication use.

## Materials and methods

### Study design and setting

The PSYKIM study was a large cross-sectional survey investigating the use of CIM among adults with psychiatric disorders in Germany. Ethical approval was obtained from the Charité ethics committee (EA2/058/23), and the study was registered in the German Clinical Trials Registry (DRKS00032426). All procedures complied with the Declaration of Helsinki and Good Clinical Practice (ICH-GCP) guidelines. The study was coordinated by the Institute of Social Medicine, Epidemiology and Health Economics, Charité—Universitätsmedizin Berlin. Further methodological details regarding recruitment procedures, questionnaire development, and additional study outcomes are reported elsewhere.

### Participants and recruitment

Adults with self-reported psychiatric diagnoses were recruited from ambulatory psychiatric, psychosomatic, and psychotherapeutic care settings in Berlin and Brandenburg, as well as through a nationwide anonymous online survey disseminated via mental health organizations, self-help groups, and university networks. Eligible participants were adults aged 18 years or older who voluntarily and anonymously participated after providing informed consent.

### Survey instrument

The structured PSYKIM questionnaire assessed sociodemographic characteristics, psychiatric diagnoses, symptom course, CIM use and evaluation, physician awareness of CIM use, perceived effectiveness of CIM approaches, conventional medication use, and economic aspects related to CIM use.

The present analyses focused on three questionnaire items assessing financial burden related to CIM treatments used for psychiatric complaints:

- whether participants had personally paid out-of-pocket costs for CIM use (yes/no),
- the approximate amount spent on CIM during the previous month (≤ €50, €51–100, €101–200, >€200), and
- the amount of disposable monthly income available after living expenses (<€50, €51–100, €101–200, €201–500, €501–1,000, €1,001–1,500, >€1,500).

For participants reporting multiple CIM approaches, one overall evaluation category was assigned at the participant level. This category reflected the evaluation reported most often across the approaches used by that participant. This approach was chosen to retain one observation per participant in the analyses. If two or more categories were reported equally often, one of the tied categories was selected randomly.

### Statistical analysis

Data were analyzed using R (version 4.5.3). All analyses followed a cross-sectional design. Prior to analysis, plausibility and completeness checks were performed to ensure data quality. Descriptive statistics were used to summarize the data; continuous variables are reported as means with standard deviations or medians with interquartile ranges (IQR), and categorical variables as frequencies and percentages. A ratio of out-of-pocket expenses to monthly disposable income was approximated by recoding categorical response options to midpoint values and dividing the estimated out-of-pocket expenses by the estimated monthly disposable income. This ratio was reported descriptively by relevant grouping variables as median and IQR.

Associations between explanatory variables and cost-related outcomes were examined using multivariable regression models. Binary logistic regression was used for the outcome indicating whether any CIM-related costs were paid out of pocket, and ordinal logistic regression was used for ordered out-of-pocket cost categories. The multivariable models included monthly disposable income, disease duration, age, sex, evaluation of CIM approaches, physician awareness of CIM use, and health insurance status simultaneously. Results are reported as adjusted odds ratios (aORs) with 95% confidence intervals. Model assumptions were checked by inspecting sparse categories, assessing multicollinearity among predictors, and testing the proportional odds assumption for ordinal logistic regression models using the Brant test.

As a sensitivity analysis, separate regression models were fitted for each explanatory variable and adjusted for age and sex. Educational level and primary psychiatric diagnosis were described descriptively but were not included in the multivariable models because of sparse categories.

The association between CIM use and conventional medication use was analyzed using binary logistic regression adjusted for age and sex. Current CIM use was defined as use of at least one listed CIM approach. Conventional medication use was defined as present if participants indicated current use of conventional medication and provided a corresponding medication entry.

## Results

### Sample characteristics

A total of 768 participants were included between June 2023 and August 2024. Participants were predominantly female (77.3%) and had a mean age of 43.2 years (SD = 14.4). Participants lived in Berlin (30.5%), Brandenburg (31.5%), or other German federal states (38.0%). Recruitment took place online (44.3%) or in ambulatory/on-site settings (55.4%).

Most participants reported a recurrent course of illness (82.3%) and symptom duration of more than 6 months (81.9%). Overall, 26.6% were currently on sick leave due to psychiatric disorders. Educational background and employment status were heterogeneous. Most participants lived with others in their household (63.7%). Detailed sociodemographic characteristics are shown in Table 1.

**Table 1:** Sociodemographic characteristics.

Characteristic	Total sample (*N* = 768)	Participants with complete cost data (*N* = 657)
Sex		
Female	594 (77.3%)	524 (79.8%)
Male	164 (21.4%)	123 (18.7%)
Diverse	10 (1.3%)	10 (1.5%)
Age	43.2 (SD: 14.4)	42.8 (SD: 14.2)
Missing	*n =* 2	*n =* 1
Place of residence (Federal State)		
Berlin	234 (30.5%)	200 (30.5%)
Brandenburg	241 (31.5%)	179 (27.3%)
Other federal states	291 (38.0%)	276 (42.1%)
Missing	*n =* 2	*n =* 2
Education		
Vocational apprenticeship	166 (21.7%)	134 (20.4%)
Vocational school	71 (9.3%)	60 (9.1%)
Technical school	65 (8.5%)	55 (8.4%)
University of applied sciences	82 (10.7%)	73 (11.1%)
University	246 (32.2%)	231 (35.2%)
Other vocational qualification	19 (2.5%)	17 (2.6%)
Currently in vocational training	48 (6.3%)	40 (6.1%)
No vocational qualification	67 (8.8%)	46 (7.0%)
Missing	*n =* 4	*n =* 1
Current employment		
Employed/Civil servant	338 (44.1%)	297 (45.2%)
Self-employed	51 (6.6%)	48 (7.3%)
Unemployed	71 (9.3%)	56 (8.5%)
Studying	67 (8.7%)	60 (9.1%)
Retired	184 (24.0%)	150 (22.8%)
Other	45 (5.9%)	36 (5.5%)
Pupil/In training	11 (1.4%)	10 (1.5%)
Missing	*n =* 1	*n =* 0
Health insurance		
Private insurance	51 (6.7%)	41 (6.2%)
Statutory insurance	708 (92.4%)	610 (92.8%)
Other insurance	7 (0.9%)	6 (0.9%)
No insurance	0 (0%)	0 (0.0%)
Missing	*n =* 2	*n =* 0
People in household		
Living alone	278 (36.3%)	234 (35.7%)
Living with others	487 (63.7%)	421 (64.3%)
Missing	*n =* 3	*n =* 2
Currently on sick leave due to psychiatric disorders		
No	557 (73.4%)	490 (75.2%)
Yes	202 (26.6%)	162 (24.8%)
Missing	*n =* 9	*n =* 5
Currently retired early due to psychiatric disorders		
No	634 (83.0%)	548 (83.8%)
Yes	130 (17.0%)	106 (16.2%)
Missing	*n =* 4	*n =* 3

Values are presented as *n* (%) except for age, which is presented as mean (SD). Percentages are based on valid responses; missing values are reported separately.

The most frequently reported primary psychiatric disorders were depression (45.6%), reactions to severe stress (16.2%), anxiety disorders (10.7%), and personality disorders (5.6%). Further information on the use and evaluation of CIM, as well as further analyses, is provided in the paper “Use of Complementary and Integrative Medicine for Mental Disorders: a detailed report of this study will be published elsewhere (Siewert et al.).

Of the total sample, 657 participants provided complete data on out-of-pocket costs and were included in the cost-related analyses; their sociodemographic characteristics were broadly comparable to those of the total sample (Table 1). Among these participants, 71.5% reported out-of-pocket CIM-related costs. Most participants reported low monthly costs, although some participants reported substantial expenditures. The mean cost-to-disposable-income ratio was 0.33 (SD = 1.04), with a median of 0.07 (IQR: 0.00–0.21), indicating a skewed distribution with higher relative burden in some participants.

### Associations

Detailed descriptive tables and regression results for all examined associations are provided in the Appendix. These include descriptive associations with the proportion of participants reporting out-of-pocket costs (Supplementary Table S1), out-of-pocket cost categories (Supplementary Table S2), the cost-to-disposable-income ratio (Supplementary Table S3). The multivariable binary and ordinal logistic regression models are shown in Supplementary Tables S4, S5. Exploratory separate regression models adjusted for age and sex are provided as sensitivity analyses in Supplementary Table S6 and Table S7. No substantial violations of the model assumptions were observed. Due to the cross-sectional design, the direction of associations cannot be determined.

### Variables associated with costs

#### Disposable income

Monthly disposable income was positively associated with the likelihood of reporting out-of-pocket costs, with the proportion increasing from 65.2% among participants with less than €50 available per month to 86.2% among those with more than €1,500 (aOR = 1.19; 95% CI: 1.04–1.37; *p* = 0.014).

In addition, higher disposable income was associated with higher categories of out-of-pocket costs (aOR = 1.18; 95% CI: 1.06–1.31; *p* = 0.002). At the same time, the relative proportion of expenditures in relation to available income decreased as disposable income increased.

#### Physician CIM awareness

Participants whose treating physician was aware of their use of CIM treatments were more likely to report out-of-pocket costs, with proportions increasing from 61.7% among those whose physician was unaware to 83.5% among those whose physician was informed (aOR = 2.26; 95% CI: 1.45–3.55; *p* < 0.001). In addition, physician awareness was associated with higher cost categories (aOR = 1.59; 95% CI: 1.12–2.26; *p* = 0.010), while the relative proportion of expenditures in relation to available income was slightly higher (median 0.1 vs. 0.0).

#### Evaluation of CIM approaches

More positive evaluations of the treatments were associated with out-of-pocket costs, with the proportion of participants reporting out-of-pocket costs increasing from 41.7% among those reporting worsening and 62.6% among those reporting no improvement to 76.6% among those reporting some improvement and 80.7% among those reporting improvement (aOR = 2.09; 95% CI: 1.53–2.89; *p* < 0.001).

In addition, more positive evaluations were associated with higher cost categories (aOR = 1.88; 95% CI: 1.49–2.38; *p* < 0.001). The relative proportion of expenditures in relation to available income remained generally low and showed no consistent pattern across evaluation groups.

#### Sex, age, and educational level

Men less frequently reported out-of-pocket costs than women (58.5% vs. 74.6%; aOR = 0.44; 95% CI: 0.26–0.76; *p* = 0.003). Men also had lower odds of being in higher cost categories (aOR = 0.62; 95% CI: 0.40–0.96; *p* = 0.033), and the relative proportion of expenditures was lower among men (median 0.0 vs. 0.1). Age was not associated with reporting any out-of-pocket costs or higher odds of being in a higher cost category.

In descriptive analyses, cost patterns also differed by educational level. Participants with university or university of applied sciences degrees more often reported out-of-pocket costs (81.4% and 80.8%, respectively), whereas this proportion was lowest among participants without completed vocational training (45.7%). The relative proportion of expenditures remained low across educational groups and showed no clear consistent pattern.

### Variables not associated with costs

#### Disease duration

Disease duration was not associated with out-of-pocket costs: participants with a disease duration of more than 6 months reported out-of-pocket costs at similar rates as those with a shorter duration (71.6% vs. 70.4%; aOR = 0.86; 95% CI: 0.36–1.85; *p* = 0.710). Likewise, disease duration was not associated with higher cost categories (aOR = 1.11; 95% CI: 0.63–1.95; *p* = 0.724). The relative proportion of expenditures in relation to available income was comparable between groups (median 0.1 in both groups), although participants with longer disease duration were somewhat more frequently represented in the highest cost category (>€200).

#### Health insurance

Health insurance status was not associated with out-of-pocket costs. Participants with statutory and private health insurance reported out-of-pocket costs at similar rates (71.6% vs. 70.7%), and no differences were observed in the odds of reporting out-of-pocket costs (aOR = 0.65; 95% CI: 0.25–1.86; *p* = 0.393) or higher cost categories (aOR = 0.76; 95% CI: 0.37–1.56; *p* = 0.456) for privately insured participants compared with those with statutory insurance. Across insurance groups, expenditures were most commonly reported in the lower cost categories, while the relative proportion of expenditures in relation to available income was slightly higher among participants with statutory than private insurance (median 0.1 vs. 0.0).

#### Primary psychiatric disorders

The proportion of participants reporting out-of-pocket costs was broadly similar across the largest diagnostic groups, including depression (73.6%), anxiety disorders (76.1%), and reactions to severe stress (70.1%). More pronounced variation was observed mainly in smaller diagnostic subgroups and should therefore be interpreted with caution. Across diagnostic groups, expenditures were most commonly reported in the lower cost categories, and higher cost categories showed no clear diagnosis-related pattern. The relative financial burden remained generally low across diagnoses.

### CIM use and conventional medication

The proportion of participants using non-CIM medication was similar among CIM users and non-users (36.9% vs. 31.5%). In exploratory binary logistic regression adjusted for age and sex, CIM use was not associated with the odds of using non-CIM medication (aOR = 1.26; 95% CI: 0.91–1.76; *p* = 0.171).

### Sensitivity analysis

Overall, the exploratory models only adjusted for age and sex showed patterns largely consistent with the multivariable main analyses. Higher disposable income, physician awareness of CIM use, female sex, and more positive evaluation of CIM approaches were associated with out-of-pocket costs and/or higher cost categories. Disease duration and health insurance status were not associated with either outcome. Age was associated with higher cost categories in the exploratory ordinal models, but not in the multivariable ordinal model.

## Discussion

Overall, the findings suggest that the use of CIM among individuals with mental disorders is frequently associated with private out-of-pocket expenditures and appears to be influenced by socioeconomic gradients. Higher income was associated with a greater likelihood of self-financed CIM use. In unadjusted descriptive analyses, participants with higher educational attainment also more frequently reported self-financed CIM use. Taken together, these findings may indicate socioeconomic differences in access to CIM-related services, although the findings regarding education should be interpreted cautiously. In contrast, duration of illness was not associated with self-financed expenditures, which may reflect selection effects within a particularly health-engaged study population. The findings provide no indication that CIM use was associated with lower use of conventional medication. However, substitution of other forms of conventional mental healthcare could not be assessed. Positive subjective evaluations of CIM and physician awareness of CIM use were both associated with higher self-financed expenditures, potentially reflecting greater willingness to invest in perceived beneficial treatments and more open patient–physician communication regarding CIM use.

## Strengths and limitations

This study has several strengths. To our knowledge, it is among the few studies examining out-of-pocket expenditures for CIM among individuals with mental disorders from a health economic perspective. The relatively large sample size and the inclusion of different mental disorders allowed for the exploration of socioeconomic patterns, expenditure categories, and associations with subjective evaluations and physician awareness. In addition, the study provides insight into real-world CIM use and self-financed healthcare behavior in a population for which economic aspects of CIM utilization remain insufficiently studied. To reduce potential selection effects related to digital participation, individuals with limited digital literacy could also participate through on-site recruitment. Furthermore, recruitment sites were selected using randomized procedures based on lists obtained from statutory health insurance associations, including outpatient practices, day clinics, and other psychiatric care settings, thereby aiming to improve the diversity and representativeness of the sample.

Several limitations should also be considered. First, the cross-sectional design does not allow conclusions regarding causality or the temporal direction of associations for example, the temporal direction between expenditures and subjective evaluations of CIM remains unclear. Second, all data were self-reported and may therefore be affected by recall bias and reporting bias. Third, despite efforts to broaden recruitment, the study may still be subject to selection effects, as individuals with greater interest in CIM or more active health-seeking behavior may have been more likely to participate. Finally, missing data for some variables may have introduced additional uncertainty into the analyses.

### Socioeconomic inequalities and access to CIM

The interpretation of CIM-related expenditures must be considered within the context of national healthcare systems, as the definition, integration, and reimbursement of CIM vary substantially across countries. While some CIM procedures are integrated into routine care or partially reimbursed in certain healthcare systems, many treatments in Germany remain primarily privately financed or are covered only as optional statutory insurance benefits.

The findings of this study suggest that CIM use among individuals with mental disorders is associated with socioeconomic factors and shows socioeconomic patterning in out-of-pocket expenditures. Participants with higher available income were more likely to report CIM-related expenditures and higher expenditure categories, although the relative financial burden decreased with increasing income. These findings are consistent with previous European research demonstrating that CIM use is socially patterned and associated with greater financial and educational resources (9). Similar observations have been reported in studies from the United States, where out-of-pocket expenditures for CIM were found to be concentrated among a relatively small group of users with greater financial capacity (13).

Given that many CIM procedures are not routinely reimbursed within statutory health insurance and often require private payment, the observed association between income and CIM-related expenditures may reflect differences in financial capacity to purchase CIM services rather than differences in healthcare needs alone. No consistent differences in expenditure patterns across diagnostic groups were observed.

The observed socioeconomic gradient is particularly relevant in the context of mental disorders, where unmet care needs and barriers to psychotherapeutic treatment are common and may contribute to interest in additional treatment options (14). Higher expenditures were also associated with physician awareness of CIM use and more positive evaluations of CIM, suggesting greater engagement with CIM-related care. The higher prevalence of self-financed expenditures among women may reflect previously described gender differences in CIM utilization, as women have consistently been found to engage more frequently with CIM healthcare approaches across European populations (15).

Duration of illness was not associated with the likelihood or extent of self-financed CIM expenditures. This finding is notable given that chronic illnesses are commonly associated with persistent out-of-pocket healthcare expenditures and ongoing financial burden related to long-term treatment and disease management (12, 16–18).

One possible explanation may be that individuals with greater interest in CIM or more proactive health-seeking behavior were more likely to participate regardless of illness duration. Overall, the findings suggest that financial resources may play a role in shaping CIM utilization and expenditure patterns among individuals with mental disorders, highlighting the importance of considering equity aspects in future discussions on integrative mental healthcare.

### CIM as an additive rather than substitutive form of care

The findings of this study suggest that CIM is primarily used as an adjunct rather than a substitute for conventional psychiatric treatment. The use of CIM was not associated with lower use of non-CIM medication, suggesting that participants did not appear to replace conventional medication with CIM therapies.

This interpretation is consistent with previous research indicating that individuals with mental health disorders frequently use both conventional healthcare and CIM. This includes prescription medications as well as CIM products, practitioners, and practices. Rather than indicating a clear substitution of conventional treatment, these patterns suggest that conventional healthcare and CIM are commonly used together (12).

In this context, CIM use may reflect attempts to manage persistent symptoms, seek additional forms of support, or complement conventional mental healthcare (10). At the same time, additive use of CIM may contribute to an overall increase in healthcare-related expenditures rather than reducing costs through substitution of conventional care.

Interestingly, physician awareness of CIM use was associated with higher odds of self-financed expenditures and higher expenditure categories. Previous research has highlighted the importance of physician–patient communication and disclosure regarding CIM use within integrative care settings, suggesting that disclosure behavior may be shaped by the quality and openness of patient–provider communication (19, 20). One possible explanation is that more open communication may facilitate disclosure and integration of CIM use within ongoing treatment contexts. Alternatively, physician awareness may reflect greater healthcare engagement among participants willing to invest financially in CIM treatments. From a clinical perspective, these findings highlight the importance of discussing CIM use in routine psychiatric care. Open patient–physician communication may facilitate the safe integration of CIM alongside conventional treatment. Given the cross-sectional design, causal interpretations remain limited. Nevertheless, the findings support the notion that CIM use among individuals with mental disorders is embedded within broader patterns of healthcare utilization rather than representing a clear alternative to conventional psychiatric treatment.

### Willingness-to-pay and perceived benefit

Positive subjective evaluations of CIM were associated with both a higher likelihood of self-financed expenditures and higher expenditure categories. Participants who reported that CIM had helped or somewhat helped more frequently reported out-of-pocket expenditures than those reporting no improvement or worsening of symptoms. One possible interpretation is that individuals perceiving a benefit from CIM may be more likely to continue using and privately financing these treatments, particularly in the context of mental disorders where unmet therapeutic needs and persistent symptom burden are common. Previous research has similarly reported associations between perceived helpfulness of CIM, positive treatment beliefs, and willingness to pay for CIM treatments, particularly among individuals experiencing persistent symptom burden (21).

At the same time, the direction of this association remains unclear. Higher expenditures may be associated with greater perceived benefit, but individuals who invest financially in CIM may also evaluate these treatments more positively in retrospect. Such associations may partly reflect treatment expectations or post-decision justification. Due to the cross-sectional design, no conclusions regarding causality or temporal sequence can be drawn.

From a health economic perspective, the findings suggest that subjective treatment evaluations are associated with healthcare utilization and private spending patterns in the context of CIM use, even in the absence of routine insurance coverage.

## Conclusion

The findings of this study suggest that the use of CIM among individuals with mental disorders in Germany is frequently associated with private out-of-pocket expenditures and socioeconomic patterning in self-financed CIM use. Higher income was associated with a greater likelihood of self-financed CIM use. In unadjusted descriptive analyses, participants with higher educational attainment also more frequently reported self-financed CIM use. These findings may indicate socioeconomic differences in access to CIM healthcare services, although the descriptive findings regarding education require cautious interpretation. At the same time, CIM use was not associated with lower use of conventional medication, supporting the interpretation that CIM is primarily used alongside rather than instead of conventional psychiatric treatment.

Positive subjective evaluations of CIM and physician awareness of CIM use were also associated with higher self-financed expenditures. However, due to the cross-sectional design, no conclusions regarding causality or temporal direction can be drawn. Therefore, these findings should be critically evaluated in a prospective observational study.

Overall, the findings highlight the relevance of considering economic and equity-related aspects of CIM use in mental healthcare settings. Future longitudinal and health economic studies are needed to better understand patterns of CIM-related expenditures, potential access barriers, and the role of CIM treatments within integrated models of mental healthcare.

## Data Availability

The raw data supporting the conclusions of this article will be made available by the corresponding author upon reasonable request.

## References

[1] Arias DS. Saxena, Verguet S. Quantifying the global burden of mental disorders and their economic value. EClinicalMedicine. (2022) 54:101675. doi: 10.1016/j.eclinm.2022.101675 36193171 PMC9526145

[2] Christensen MK Lim CCW Saha S Plana-Ripoll O Cannon D Presley F . The cost of mental disorders: a systematic review. Epidemiol Psychiatr Sci. (2020) 29:e161. doi: 10.1017/S204579602000075X 32807256 PMC7443800

[3] Jacobi F Höfler M Siegert J Mack S Gerschler A Scholl L . Twelve-month prevalence, comorbidity and correlates of mental disorders in Germany: the mental health module of the German health interview and examination survey for adults (DEGS1-MH). Int J Methods Psychiatr Res. (2014) 23:304–19. doi: 10.1002/mpr.1439 24729411 PMC6878234

[4] König H König HH Gallinat J Lambert M Karow A Peth J . Excess costs of mental disorders by level of severity. Soc Psychiatry Psychiatr Epidemiol. (2023) 58:973–85. doi: 10.1007/s00127-022-02298-8 35639134 PMC10241728

[5] Kocis Krutilova VL Bahnsen De Graeve D. Krutilova The out-of-pocket burden of chronic diseases: the cases of Belgian, Czech and German older adults. BMC Health Serv Res. (2021) 21:239. doi: 10.1186/s12913-021-06259-w 33731090 PMC7967967

[6] Klein JD Ludecke O. von dem Knesebeck. Forgone and delayed care in Germany- inequalities and perceived health risk of unmet need. Int J Equity Health. (2025) 24:122. doi: 10.1186/s12939-025-02483-6 40329292 PMC12057108

[7] Jeitler M Ortiz M Brinkhaus B Sigl M Hoffmann R Trübner M . Use and acceptance of traditional, complementary and integrative medicine in Germany-an online representative cross-sectional study. Front Med. (2024) 11:1372924. doi: 10.3389/fmed.2024.1372924 38545512 PMC10965565

[8] Lederer AK Samstag Y Simmet T Syrovets T Huber R. Complementary medicine usage in surgery: a cross-sectional survey in Germany. BMC Complement Med Ther. (2022) 22:263. doi: 10.1186/s12906-022-03746-3 36221070 PMC9552450

[9] Fjær EL Landet ER McNamara CL Eikemo TA. The use of complementary and alternative medicine (CAM) in Europe. BMC Complement Med Ther. (2020) 20:108. doi: 10.1186/s12906-020-02903-w 32252735 PMC7137515

[10] de Jonge P Wardenaar KJ Hoenders HR Evans-Lacko S Kovess-Masfety V Aguilar-Gaxiola S . Complementary and alternative medicine contacts by persons with mental disorders in 25 countries: results from the World Mental Health Surveys. Epidemiol Psychiatr Sci. (2018) 27:552–67. doi: 10.1017/S2045796017000774 29283080 PMC6849470

[11] Purohit MP Zafonte RD Sherman LM Davis RB Giwerc MY Shenton ME . Neuropsychiatric symptoms and expenditure on complementary and alternative medicine. J Clin Psychiatry. (2015) 76:e870–6. doi: 10.4088/JCP.13m08682 26231014 PMC4729567

[12] McIntyre E Oorschot T Steel A Leach MJ Adams J Harnett J. Conventional and complementary health care use and out-of-pocket expenses among Australians with a self-reported mental health diagnosis: a cross-sectional survey. BMC Health Serv Res. (2021) 21:1266. doi: 10.1186/s12913-021-07162-0 34814916 PMC8611990

[13] Davis MA Weeks WB. The concentration of out-of-pocket expenditures on complementary and alternative medicine in the United States. Altern Ther Health Med. (2012) 18:36–42. 22894889 PMC3523202

[14] Birgel V Gellert P Hölling H Kuhnert R Michalski N Rapp M . Barriers to accessing psychotherapeutic care among young adults: Individual and regional associated factors J Health Monit. (2025) 10:e13549. 41356150 10.25646/13549 PMC12679805

[15] Kemppainen LM Kemppainen TT. Changes in Complementary and Alternative Medicine (CAM) use from 2014 to 2023: findings from a cross-national population-based survey in Europe. J Public Health. (2025). doi: 10.1007/s10389-025-02494-1

[16] Cunningham PJ. Chronic burdens: the persistently high out-of-pocket health care expenses faced by many Americans with chronic conditions. Issue Brief. (2009) 63:1–14. 19626725

[17] Essue B Laba M Knaul F Chu A Minh HV Nguyen TKP . Economic burden of chronic ill-health and injuries for households in low-and middle-income countries. Disease control priorities: improving health and reducing poverty. 3rd ed. Washington (DC): The International Bank for Reconstruction and Development/The World Bank; 2017 (2018). 30212160

[18] Callander EJ Corscadden L Levesque JF. Out-of-pocket healthcare expenditure and chronic disease - do Australians forgo care because of the cost? Aust J Prim Health. (2017) 23:15–22. doi: 10.1071/PY16005 28442033

[19] Foley H Steel A McIntyre E Harnett J Sibbritt D Adams J. Disclosure of conventional and complementary medicine use to medical doctors and complementary medicine practitioners: A survey of rates and reasons amongst those with chronic conditions. PLoS ONE. (2021) 16:e0258901. doi: 10.1371/journal.pone.0258901 34735474 PMC8568289

[20] Foley H Steel A Cramer H Wardle J Adams J. Disclosure of complementary medicine use to medical providers: a systematic review and meta-analysis. Sci Rep. (2019) 9:1573. doi: 10.1038/s41598-018-38279-8 30733573 PMC6367405

[21] Montross-Thomas LP Meier EA Reynolds-Norolahi K Raskin EE Slater D Mills PJ . Inpatients' preferences, beliefs, and stated willingness to pay for complementary and alternative medicine treatments. J Altern Complement Med. (2017) 23:259–63. doi: 10.1089/acm.2016.0288 28112554

