# Supplementary material for: Complementary and integrative medicine use in mental disorders: socioeconomic patterns and healthcare utilization in Germany
Source: Front Med (Lausanne). 2026 Aug 21;13:1904690. doi: 10.3389/fmed.2026.1904690 (PMC13541536; doi:10.3389/fmed.2026.1904690)
Supplement: Supplementary file 1 [file Data_Sheet_1.pdf]

Appendix: Supplementary Tables

Out-of-pocket costs, cost categories, relative financial burden, and regression results

Contents

- Table S1. Descriptive associations with out-of-pocket costs
- Table S2. Descriptive associations with cost categories
- Table S3. Descriptive associations with relative financial burden
- Table S4. Multivariable binary logistic regression
- Table S5. Multivariable ordinal logistic regression
- Table S6. Age- and sex-adjusted binary logistic regression, sensitivity analysis
- Table S7. Age- and sex-adjusted ordinal logistic regression, sensitivity analysis

Notes: Percentages are calculated among participants with valid data for the respective outcome. Missing refers to missing outcome data within the category. Relative financial burden is the approximated cost-to-disposable-income ratio based on class midpoints and is presented descriptively only.

Table S1. Descriptive associations with out-of-pocket costs

Outcome: any self-paid costs for CIM approaches in the previous month.

Variable	Category	n total	No	Yes	Missing
Monthly disposable income	Under 50 EUR	47	16 (34.8%)	30 (65.2%)	1
	51-100 EUR	53	13 (24.5%)	40 (75.5%)	0
	101-200 EUR	92	21 (23.9%)	67 (76.1%)	4
	201-500 EUR	179	54 (31.4%)	118 (68.6%)	7
	501-1000 EUR	121	29 (24.6%)	89 (75.4%)	3
	1001-1500 EUR	77	16 (21.3%)	59 (78.7%)	2
	More than 1500 EUR	65	9 (13.8%)	56 (86.2%)	0
Primary psychiatric disorder	Depression	350	79 (26.4%)	220 (73.6%)	51

Variable	Category	n total	No	Yes	Missing
	Other mood disorder	27	10 (40.0%)	15 (60.0%)	2
	Anxiety disorder	82	17 (23.9%)	54 (76.1%)	11
	Eating disorder	19	4 (28.6%)	10 (71.4%)	5
	Personality disorder	43	19 (51.4%)	18 (48.6%)	6
	Schizophrenia	17	4 (33.3%)	8 (66.7%)	5
	Other psychotic/delusional disorder	4	0 (0.0%)	3 (100.0%)	1
	Substance use disorder (alcohol/drugs)	12	3 (33.3%)	6 (66.7%)	3
	Substance use disorder (nicotine)	3	0 (0.0%)	3 (100.0%)	0
	Obsessive-compulsive disorder	22	4 (18.2%)	18 (81.8%)	0
	Psychosomatic disorder	22	4 (20.0%)	16 (80.0%)	2
	Reaction to severe stress	124	32 (29.9%)	75 (70.1%)	17
	Dementia	2	0 (0.0%)	1 (100.0%)	1
	Other psychiatric diagnosis	4	1 (25.0%)	3 (75.0%)	0
	Unknown psychiatric diagnosis	9	1 (14.3%)	6 (85.7%)	2
	ADHD	13	3 (25.0%)	9 (75.0%)	1
	Autism spectrum disorder	4	3 (75.0%)	1 (25.0%)	0
	Mixed anxiety and depressive disorder	10	3 (50.0%)	3 (50.0%)	4
Disease duration	<6 months	67	16 (29.6%)	38 (70.4%)	13
	>6 months	627	153 (28.4%)	385 (71.6%)	89

Variable	Category	n total	No	Yes	Missing
Sex	Female	594	133 (25.4%)	391 (74.6%)	70
	Male	164	51 (41.5%)	72 (58.5%)	41
	Diverse	10	3 (30.0%)	7 (70.0%)	0
Education	Vocational apprenticeship	166	55 (41.0%)	79 (59.0%)	32
	Vocational school	71	19 (31.7%)	41 (68.3%)	11
	Technical school	65	12 (21.8%)	43 (78.2%)	10
	University of applied sciences	82	14 (19.2%)	59 (80.8%)	9
	University	246	43 (18.6%)	188 (81.4%)	15
	Other educational qualification	19	4 (23.5%)	13 (76.5%)	2
	Currently in vocational training	48	15 (37.5%)	25 (62.5%)	8
	No completed vocational training	67	25 (54.3%)	21 (45.7%)	21
Evaluation of CIM approaches	Worsened	12	7 (58.3 %)	5 (41.7 %)	2
	No help	190	71 (37.4 %)	119 (62.6 %)	10
	Some help	290	68 (23.4 %)	222 (76.6 %)	24
	Helped	140	27 (19.3 %)	113 (80.7 %)	9
Physician awareness of CIM use	No	233	87 (38.3%)	140 (61.7%)	6
	Yes	363	60 (16.5%)	303 (83.5%)	0
Health insurance	Statutory insurance	708	173 (28.4%)	437 (71.6%)	98
	Private insurance	51	12 (29.3%)	29 (70.7%)	10
	Other insurance	7	2 (33.3%)	4 (66.7%)	1

Table S2. Descriptive associations with cost categories

Outcome: approximate CIM-related costs in the previous month.

Variable	Category	n total	0 EUR	Up to 50 EUR	51-100 EUR	101-200 EUR	>200 EUR	Missing
Monthly disposable income	Under 50 EUR	47	18 (41.9%)	14 (32.6%)	5 (11.6%)	1 (2.3%)	5 (11.6%)	4
	51-100 EUR	53	13 (26.5%)	21 (42.9%)	7 (14.3%)	7 (14.3%)	1 (2.0%)	4
	101-200 EUR	92	21 (25.3%)	41 (49.4%)	10 (12.0%)	7 (8.4%)	4 (4.8%)	9
	201-500 EUR	179	50 (32.1%)	55 (35.3%)	19 (12.2%)	18 (11.5%)	14 (9.0%)	23
	501-1000 EUR	121	28 (26.2%)	36 (33.6%)	15 (14.0%)	12 (11.2%)	16 (15.0%)	14
	1001-1500 EUR	77	18 (25.7%)	23 (32.9%)	12 (17.1%)	5 (7.1%)	12 (17.1%)	7
	More than 1500 EUR	65	14 (23.0%)	14 (23.0%)	12 (19.7%)	10 (16.4%)	11 (18.0%)	4
Primary psychiatric disorder	Depression	350	69 (25.8%)	103 (38.6%)	42 (15.7%)	32 (12.0%)	21 (7.9%)	83
	Other mood disorder	27	9 (42.9%)	7 (33.3%)	1 (4.8%)	1 (4.8%)	3 (14.3%)	6
	Anxiety disorder	82	18 (27.7%)	21 (32.3%)	8 (12.3%)	10 (15.4%)	8 (12.3%)	17
	Eating disorder	19	5 (35.7%)	3 (21.4%)	1 (7.1%)	1 (7.1%)	4 (28.6%)	5
	Personality disorder	43	14 (46.7%)	10 (33.3%)	3 (10.0%)	0 (0.0%)	3 (10.0%)	13
	Schizophrenia	17	0 (0.0%)	8 (100.0%)	0 (0.0%)	0 (0.0%)	0 (0.0%)	9
	Other psychotic/delusional disorder	4	0 (0.0%)	3 (100.0%)	0 (0.0%)	0 (0.0%)	0 (0.0%)	1
	Substance use disorder (alcohol/drugs)	12	2 (28.6%)	2 (28.6%)	0 (0.0%)	1 (14.3%)	2 (28.6%)	5
	Substance use disorder (nicotine)	3	0 (0.0%)	1 (33.3%)	0 (0.0%)	1 (33.3%)	1 (33.3%)	0

Variable	Category	n total	0 EUR	Up to 50 EUR	51-100 EUR	101-200 EUR	>200 EUR	Missing
	Obsessive-compulsive disorder	22	8 (38.1%)	5 (23.8%)	3 (14.3%)	0 (0.0%)	5 (23.8%)	1
	Psychosomatic disorder	22	2 (11.8%)	5 (29.4%)	6 (35.3%)	3 (17.6%)	1 (5.9%)	5
	Reaction to severe stress	124	29 (31.2%)	25 (26.9%)	16 (17.2%)	10 (10.8%)	13 (14.0%)	31
	Dementia	2	0 (0.0%)	1 (100.0%)	0 (0.0%)	0 (0.0%)	0 (0.0%)	1
	Other psychiatric diagnosis	4	0 (0.0%)	2 (66.7%)	0 (0.0%)	1 (33.3%)	0 (0.0%)	1
	Unknown psychiatric diagnosis	9	0 (0.0%)	4 (66.7%)	1 (16.7%)	1 (16.7%)	0 (0.0%)	3
	ADHD	13	4 (33.3%)	6 (50.0%)	0 (0.0%)	0 (0.0%)	2 (16.7%)	1
	Autism spectrum disorder	4	3 (75.0%)	0 (0.0%)	1 (25.0%)	0 (0.0%)	0 (0.0%)	0
	Mixed anxiety and depressive disorder	10	0 (0.0%)	2 (66.7%)	0 (0.0%)	0 (0.0%)	1 (33.3%)	7
Disease duration	<6 months	67	12 (25.5%)	21 (44.7%)	8 (17.0%)	5 (10.6%)	1 (2.1%)	20
	>6 months	627	133 (27.9%)	169 (35.4%)	68 (14.3%)	48 (10.1%)	59 (12.4%)	150
Sex	Female	594	125 (26.7%)	163 (34.8%)	68 (14.5%)	54 (11.5%)	58 (12.4%)	126
	Male	164	35 (34.7%)	41 (40.6%)	12 (11.9%)	7 (6.9%)	6 (5.9%)	63
	Diverse	10	3 (30.0%)	4 (40.0%)	2 (20.0%)	0 (0.0%)	1 (10.0%)	0
Education	Vocational apprenticeship	166	28 (28.3%)	37 (37.4%)	11 (11.1%)	8 (8.1%)	15 (15.2%)	67
	Vocational school	71	19 (36.5%)	9 (17.3%)	8 (15.4%)	11 (21.2%)	5 (9.6%)	19
	Technical school	65	13 (25.5%)	17 (33.3%)	5 (9.8%)	8 (15.7%)	8 (15.7%)	14
	University of applied sciences	82	13 (19.1%)	27 (39.7%)	9 (13.2%)	9 (13.2%)	10 (14.7%)	14
	University	246	48 (22.1%)	84 (38.7%)	40 (18.4%)	25 (11.5%)	20 (9.2%)	29
	Other educational qualification	19	2 (14.3%)	5 (35.7%)	3 (21.4%)	0 (0.0%)	4 (28.6%)	5

Variable	Category	n total	0 EUR	Up to 50 EUR	51-100 EUR	101-200 EUR	>200 EUR	Missing
	Currently in vocational training	48	16 (41.0%)	19 (48.7%)	2 (5.1%)	0 (0.0%)	2 (5.1%)	9
	No completed vocational training	67	24 (63.2%)	10 (26.3%)	3 (7.9%)	0 (0.0%)	1 (2.6%)	29
Evaluation of CIM approaches	Worsened	14	8 (66.7 %)	2 (16.7%	0 (0.0%)	1 (8.3 %)	1 (8.3 %)	2
	No help	200	76 (44.2 %)	54 (31.4%)	15 (8.7%)	10 (5.8 %)	17 (9.9 %)	28
	Some help	314	57 (22.0 %)	99 (38.2%)	43 (16.6%)	32 (12.4%)	28 (10.8 %)	55
	Helped	149	20 (16.0 %)	46 (36.8%)	22 (17.6%)	18 (14.4%)	19 (15.2 %)	24
Physician awareness of CIM use	No	233	77 (38.5%)	67 (33.5%)	24 (12.0%)	14 (7.0%)	18 (9.0%)	33
	Yes	363	78 (22.5%)	132 (38.0%)	54 (15.6%)	43 (12.4%)	40 (11.5%)	16
Health insurance	Statutory insurance	708	151 (28.2%)	193 (36.0%)	76 (14.2%)	57 (10.6%)	59 (11.0%)	172
	Private insurance	51	9 (24.3%)	13 (35.1%)	6 (16.2%)	4 (10.8%)	5 (13.5%)	14
	Other insurance	7	3 (50.0%)	2 (33.3%)	0 (0.0%)	0 (0.0%)	1 (16.7%)	1

Table S3. Descriptive associations with relative financial burden

Outcome: approximated cost-to-disposable-income ratio. Because the variable was derived from category midpoints, it was analysed descriptively only.

Variable	Category	n	Mean (SD)	Median [IQR]
Monthly disposable income	Under 50 EUR	43	1.98 (3.19)	1.0 [0.0; 2.0]
	51-100 EUR	49	0.64 (0.77)	0.3 [0.0; 1.0]
	101-200 EUR	83	0.31 (0.41)	0.2 [0.1; 0.3]
	201-500 EUR	156	0.16 (0.22)	0.1 [0.0; 0.2]
	501-1000 EUR	107	0.10 (0.12)	0.0 [0.0; 0.2]
	1001-1500 EUR	70	0.06 (0.07)	0.0 [0.0; 0.1]
	More than 1500 EUR	61	0.05 (0.05)	0.0 [0.0; 0.1]
Primary psychiatric disorder	Depression	262	0.29 (0.95)	0.1 [0.0; 0.2]
	Other mood disorder	21	0.09 (0.12)	0.0 [0.0; 0.2]
	Anxiety disorder	62	0.32 (0.62)	0.1 [0.0; 0.3]
	Eating disorder	14	0.19 (0.26)	0.1 [0.0; 0.3]
	Personality disorder	30	0.15 (0.25)	0.0 [0.0; 0.2]
	Schizophrenia	8	0.23 (0.33)	0.1 [0.0; 0.2]
	Other psychotic/delusional disorder	3	0.69 (0.54)	1.0 [0.5; 1.0]
	Substance use disorder (alcohol/drugs)	7	0.10 (0.12)	0.1 [0.0; 0.1]
	Substance use disorder (nicotine)	2	3.36 (3.74)	3.4 [2.0; 4.7]
	Obsessive-compulsive disorder	21	0.33 (0.72)	0.0 [0.0; 0.2]
	Psychosomatic disorder	17	0.85 (2.38)	0.2 [0.1; 0.4]

Variable	Category	n	Mean (SD)	Median [IQR]
	Reaction to severe stress	93	0.37 (1.14)	0.1 [0.0; 0.2]
	Other psychiatric diagnosis	3	0.16 (0.23)	0.0 [0.0; 0.2]
	Unknown psychiatric diagnosis	6	0.10 (0.08)	0.1 [0.0; 0.2]
	ADHD	12	0.91 (2.86)	0.1 [0.0; 0.2]
	Autism spectrum disorder	4	0.01 (0.03)	0.0 [0.0; 0.0]
	Mixed anxiety and depressive disorder	3	0.15 (0.07)	0.2 [0.1; 0.2]
Disease duration	<6 months	47	0.35 (0.95)	0.1 [0.0; 0.2]
	>6 months	468	0.34 (1.11)	0.1 [0.0; 0.2]
Sex	Female	459	0.37 (1.15)	0.1 [0.0; 0.2]
	Male	100	0.15 (0.31)	0.0 [0.0; 0.2]
	Diverse	10	0.21 (0.35)	0.1 [0.0; 0.1]
Education	Vocational apprenticeship	95	0.25 (0.49)	0.1 [0.0; 0.2]
	Vocational school	50	0.99 (2.51)	0.0 [0.0; 0.4]
	Technical school	50	0.40 (1.42)	0.1 [0.0; 0.2]
	University of applied sciences	68	0.36 (1.27)	0.1 [0.0; 0.2]
	University	215	0.21 (0.38)	0.1 [0.0; 0.2]
	Other educational qualification	13	0.52 (0.87)	0.2 [0.1; 0.3]
	Currently in vocational training	39	0.15 (0.23)	0.1 [0.0; 0.2]
	No completed vocational training	38	0.27 (0.57)	0.0 [0.0; 0.3]
Evaluation of CIM approaches	Worsened	12	0.86 (2.88)	0.0 [0.0; 0.0]
	No help	171	0.25 (0.94)	0.0 [0.0; 0.2]

Variable	Category	n	Mean (SD)	Median [IQR]
	Some help	255	0.38 (1.17)	0.1 [0.0; 0.3]
	Helped	121	0.27 (0.47)	0.1 [0.0; 0.3]
Physician awareness of CIM use	No	196	0.24 (0.81)	0.0 [0.0; 0.2]
	Yes	343	0.36 (1.07)	0.1 [0.0; 0.3]
Health insurance	Statutory insurance	526	0.35 (1.08)	0.1 [0.0; 0.2]
	Private insurance	37	0.08 (0.10)	0.0 [0.0; 0.1]
	Other insurance	6	0.07 (0.13)	0.0 [0.0; 0.1]

Table S4. Results of the multivariable binary logistic regression

Outcome: any self-paid costs for CIM approaches in the previous month (yes vs. no).

Variable	Predictor	OR	95% CI	p value
Monthly disposable income	Income category (per higher category)	1.19	1.04–1.37	0.014
Disease duration	Duration category (>6 months vs. <6 months)	0.86	0.36–1.85	0.710
Age	Age (per year)	1.01	0.99–1.02	0.380
Sex	Male vs. female	0.44	0.26–0.76	0.003
	Diverse vs. female	1.34	0.23–11.77	0.763
Evaluation of CIM approaches	Evaluation category (per higher category)	2.09	1.53–2.89	<0.001
Physician awareness of CIM use	Physician aware (yes vs. no)	2.26	1.45–3.55	<0.001
Health insurance	Private vs. statutory insurance	0.65	0.25–1.86	0.393
	Other vs. statutory insurance	0.37	0.05–3.14	0.313

Table S5. Results of the multivariable ordinal logistic regression

Outcome: ordered monthly CIM-related cost categories.

Variable	Predictor	OR	95% CI	p value
Monthly disposable income	Income category (per higher category)	1.18	1.06–1.31	0.002
Disease duration	Duration category (>6 months vs. <6 months)	1.11	0.63–1.95	0.724
Age	Age (per year)	1.01	1.00–1.02	0.195
Sex	Male vs. female	0.62	0.40–0.96	0.033
	Diverse vs. female	1.15	0.33–3.90	0.818
Evaluation of CIM approaches	Evaluation category (per higher category)	1.88	1.49–2.38	<0.001
Physician awareness of CIM use	Physician aware (yes vs. no)	1.59	1.12–2.26	0.010
Health insurance	Private vs. statutory insurance	0.76	0.37–1.56	0.456
	Other vs. statutory insurance	0.28	0.03–1.76	0.185

Table S6. Results of the binary logistic regression

Outcome: any self-paid costs (yes vs. no). Models were adjusted for age and sex unless sex was the predictor of interest.

Variable	Predictor	OR	95% CI	p value
Monthly disposable income	Income category (per higher category)	1.14	1.02-1.27	0.025
	Age	1.01	0.99-1.02	0.312
	Male vs. female	0.47	0.31-0.74	<0.001
	Diverse vs. female	0.79	0.21-3.73	0.733
Disease duration	Duration (>6 months vs. <6 months)	1.00	0.52-1.83	0.996
	Age	1.01	1.00-1.02	0.138
	Male vs. female	0.56	0.36-0.86	0.008
	Diverse vs. female	1.09	0.25-7.51	0.918
Sex / Age	Male vs. female	0.47	0.31-0.71	<0.001
	Diverse vs. female	0.81	0.22-3.80	0.763
	Age	1.01	0.99-1.02	0.320
Physician awareness of CIM use	Physician aware (yes vs. no)	3.20	2.17-4.76	<0.001
	Age	1.01	1.00-1.02	0.197
	Male vs. female	0.45	0.28-0.73	0.001
	Diverse vs. female	0.67	0.17-3.29	0.584
Evaluation of CIM approaches	Evaluation category (per higher category)	1.70	1.34–2.17	<0.001
	Age	1.00	0.99–1.02	0.457
	Male vs. female	0.46	0.30–0.71	<0.001

Variable	Predictor	OR	95% CI	p value
	Diverse vs. female	0.80	0.21–3.91	0.761
Health insurance	Private vs. statutory insurance	0.90	0.46-1.88	0.769
	Other vs. statutory insurance	0.77	0.15-5.66	0.768
	Age	1.01	0.99-1.02	0.313
	Male vs. female	0.47	0.31-0.71	<0.001
	Diverse vs. female	0.80	0.22-3.77	0.753

Table S7. Results of the ordinal logistic regression

Outcome: ordered monthly cost categories. The proportional-odds assumption was checked using the Brant test.

Variable	Predictor	OR	95% CI	p value
Monthly disposable income	Income category (per higher category)	1.15	1.05-1.27	0.002
	Age	1.02	1.01-1.03	0.002
	Male vs. female	0.56	0.38-0.83	0.004
	Diverse vs. female	0.84	0.28-2.50	0.759
Disease duration	Duration (>6 months vs. <6 months)	1.17	0.70-1.99	0.551
	Age	1.02	1.01-1.03	<0.001
	Male vs. female	0.64	0.43-0.95	0.028
	Diverse vs. female	0.97	0.27-3.30	0.955
Sex / Age	Male vs. female	0.56	0.37-0.82	0.003
	Diverse vs. female	0.77	0.25-2.32	0.639
	Age	1.02	1.01-1.03	<0.001
Evaluation of CIM approaches	Evaluation category (per higher category)	1.76	1.44–2.17	<0.001
	Age	1.02	1.01–1.03	0.004
	Male vs. female	0.56	0.37–0.84	0.005
	Diverse vs. female	0.76	0.24–2.30	0.633
Physician awareness of CIM use	Physician aware (yes vs. no)	1.82	1.32-2.52	<0.001
	Age	1.02	1.01-1.03	0.002

Variable	Predictor	OR	95% CI	p value
	Male vs. female	0.55	0.37-0.82	0.004
	Diverse vs. female	0.74	0.24-2.23	0.592
Health insurance	Private vs. statutory insurance	1.06	0.58-1.93	0.853
	Other vs. statutory insurance	0.39	0.07-1.88	0.242
	Age	1.02	1.01-1.03	<0.001
	Male vs. female	0.56	0.38-0.83	0.004
	Diverse vs. female	0.77	0.25-2.31	0.635
